# Sex differences in responses to antiretroviral treatment in South African HIV-infected children on ritonavir-boosted lopinavir- and nevirapine-based treatment

**DOI:** 10.1186/1471-2431-14-39

**Published:** 2014-02-12

**Authors:** Stephanie Shiau, Louise Kuhn, Renate Strehlau, Leigh Martens, Helen McIlleron, Sandra Meredith, Lubbe Wiesner, Ashraf Coovadia, Elaine J Abrams, Stephen M Arpadi

**Affiliations:** 1 Gertrude H. Sergievsky Center, College of Physicians and Surgeons, Columbia University, New York, NY USA; 2 Department of Epidemiology, Mailman School of Public Health, Columbia University, New York, NY USA; 3 Empilweni Services and Research Unit, Rahima Moosa Mother and Child Hospital, University of the Witwatersrand, Johannesburg, South Africa; 4 Department of Medicine, Division of Clinical Pharmacology, University of Cape Town, Cape Town, South Africa; 5 Institute of Infectious Disease and Molecular Medicine, University of Cape Town, Cape Town, South Africa; 6 ICAP, Mailman School of Public Health, Columbia University, New York, NY USA; 7 Department of Pediatrics, College of Physicians and Surgeons, Columbia University, New York, NY USA

**Keywords:** HIV, Children, Sex differences, Antiretroviral treatment outcomes, Pharmacokinetics

## Abstract

**Background:**

While studies of HIV-infected adults on antiretroviral treatment (ART) report no sex differences in immune recovery and virologic response but more ART-associated complications in women, sex differences in disease progression and response to ART among children have not been well assessed. The objective of this study was to evaluate for sex differences in response to ART in South African HIV-infected children who were randomized to continue ritonavir-boosted lopinavir (LPV/r)-based ART or switch to nevirapine-based ART.

**Methods:**

ART outcomes in HIV-infected boys and girls in Johannesburg, South Africa from 2005–2010 were compared. Children initiated ritonavir-boosted lopinavir (LPV/r)-based ART before 24 months of age and were randomized to remain on LPV/r or switch to nevirapine-based ART after achieving viral suppression. Children were followed for 76 weeks post-randomization and then long-term follow up continued for a minimum of 99 weeks and maximum of 245 weeks after randomization. Viral load, CD4 count, lipids, anthropometrics, drug concentrations, and adherence were measured at regular intervals. Outcomes were compared between sexes within treatment strata.

**Results:**

A total of 323 children (median age 8.8 months, IQR 5.1-13.5), including 168 boys and 155 girls, initiated LPV/r-based ART and 195 children were randomized. No sex differences in risk of virological failure (confirmed viral load >1000 copies/mL) by 156 weeks post-randomization were observed within either treatment group. Girls switched to nevirapine had more robust CD4 count improvement relative to boys in this group through 112 weeks post-randomization. In addition, girls remaining on LPV/r had higher plasma concentrations of ritonavir than boys during post-randomization visits. After a mean of 3.4 years post-randomization, girls remaining on LPV/r also had a higher total cholesterol:HDL ratio and lower mean HDL than boys on LPV/r.

**Conclusions:**

Sex differences are noted in treated HIV-infected children even at a young age, and appear to depend on treatment regimen. Future studies are warranted to determine biological mechanisms and clinical significance of these differences.

**Trial registration:**

ClinicalTrials.gov Identifier: NCT00117728

## Background

Studies of sex differences in the course of HIV infection in adults report lower HIV-1 RNA levels and higher CD4 counts in women compared to men, but similar disease progression and clinical outcomes between sexes [1,2]. Responses to antiretroviral treatment (ART), including immune reconstitution and virologic response, are also similar [3,4]. However, ART-associated complications, including hepatotoxicity, pancreatitis, as well as metabolic abnormalities and lipodystrophy are consistently reported more often in women than men [5-11]. The differential effects in ART response between sexes appear to be driven by sex-specific physiological and hormonal influences as well as by differences in drug pharmacokinetics [12,13]. Social and behavioral factors may also play a role, as rates of adherence and treatment discontinuation vary between men and women [14].

Less is known about sex differences in disease progression and response to ART among children. Differences in CD4 count and HIV-1 RNA between boys and girls have been reported in treatment-naïve HIV-infected children [15]. In treated children, one study reported lower HIV-1 RNA in girls than boys and no differences in CD4 count [16] while two observational studies reported a more rapid immunologic response to ART in girls [17,18]. Sex differences in incidence of ART-associated complications reported in children [19-23] have rarely been assessed.

A better understanding of sex differences in the pathobiology of HIV and its complications would be aided by evaluation of these differences during early childhood when biologic, social, and behavioral differences are less pronounced than in adulthood. The objective of this study was to evaluate for sex differences in response to ART in South African HIV-infected children who were randomized to continue ritonavir-boosted lopinavir (LPV/r)-based ART or switch to nevirapine-based ART.

## Methods

### Study design

In order to assess for sex differences in immunologic, virologic, anthropometric, and other responses to antiretroviral regimens, we performed a secondary analysis of data collected as part of Neverest 2, a clinical trial (ClinicalTrials.gov: NCT00117728) assessing the reuse of nevirapine in nevirapine-exposed HIV-infected children conducted from 2005 to 2010 [24,25]. Children previously exposed to single-dose nevirapine prophylaxis at birth, and <24 months of age at the time of initiation of LPV/r-based ART, were identified at Rahima Moosa Mother and Child Hospital in Johannesburg, South Africa. Those who achieved and sustained plasma HIV-1 RNA <400 copies/mL for ≥3 months during the first 12 months of treatment were eligible for randomization to either continue on LPV/r or switch to nevirapine-based ART in combination with stavudine and lamivudine. Specific treatment regimens have been previously reported [24,25]. Children were followed for 76 weeks post-randomization and then re-enrolled in extended follow up. In this analysis, we evaluated sex differences pretreatment, in the pre-randomization phase, at the time of randomization, at post-randomization study visits, and at participants’ final visit. This study was approved by the Institutional Review Boards of Columbia University (New York, New York) and the University of the Witwatersrand (Johannesburg, South Africa). Informed consent was provided by each child’s parent or guardian.

### Measurements

Socio-demographic information, medical history, weight (kg), height (cm), and blood samples (for CD4 T-cell determination and HIV-1 RNA quantity) were collected at a pretreatment visit. Subsequent visits were scheduled at 2, 4, 8, 12, and every 12 weeks thereafter up to 52 weeks until randomization and at 2, 4, 8, 16, 24, 36, 52, 64, 76 and every 12 weeks thereafter. Blood samples for plasma viral load tests by quantitative HIV-1 RNA PCR (Roche, Cobas Ampliprep Taqman V2, Branchburg, NJ) were collected at 4, 16, 24, 36, 52, 64, and 76 weeks post-randomization and for absolute CD4 count and percentages (Beckman Coulter Flow Analyzer) at 16, 24, 36, 52, 64, and 76 weeks post-randomization, and for both tests at every 12 weeks thereafter until the final study visit. Lopinavir, ritonavir, and nevirapine plasma concentrations were determined using validated LC-MS/MS assays developed in the Division of Clinical Pharmacology, Cape Town, South Africa. An AB Sciex 4000 mass spectrometer was operated at unit resolution in the multiple reaction monitoring mode. The assays were validated over the concentration range of 0.16-20 μg/ml for lopinavir, 0.10-20 μg/ml for nevirapine, and 0.04-5 μg/ml for ritonavir.

Non-fasting total cholesterol, low-density lipoprotein (LDL), high-density lipoprotein (HDL), and triglycerides in mg/dL were measured at pretreatment, time of randomization, and 36, 88, and 136 weeks post randomization (Roche, Cobas Integra 400, Branchburg, NJ). At the final study visit following an overnight fast, total cholesterol, LDL, HDL, triglycerides, C-reactive protein in mg/L, and insulin in mg/dL were measured. Venous blood glucose in mg/dL was measured with a handheld glucometer and homeostasis model assessment of insulin resistance (HOMA-IR) was calculated [26]. Classification of biochemical results has been described previously [22]. Assessments for drug-related rashes and hepatotoxicity by alanine aminotransferase (ALT) were performed at post-randomization visits [24,25].

Weight (kg) and height (cm) were measured using a digital scale and stadiometer, respectively. Weight-for-age (WAZ) and height-for-age z-scores (HAZ) were calculated using World Health Organization growth standards [27]. Underweight was defined as WAZ < −2 and stunting was defined as HAZ < −2. Circumferences at the mid-upper arm, mid-upper thigh, mid-waist, and maximum hip were measured using a flexible tape measure with a spring tension attachment. Bicep, tricep, subscapular, suprailiac, umbilical, and mid-thigh skinfolds were measured with a Harpendon caliper (Baty International, England). As previously reported, total body fat percent (%BF) was estimated by single-frequency bioimpedance analysis (BIA) (Quantum II, RJL Systems, Clinton Township, MI) [28] and children were classified as having lipodystrophy, possible lipodystrophy, or no signs of lipodystrophy by two physicians (RS, LM) [22].

All children with HIV-1 RNA >1000 copies/mL were recalled and retested within 4 weeks. Additional counseling was provided if adherence difficulties were detected. Children in the switch group were returned to the LPV/r-based regimen if HIV-1 RNA remained >1000 copies/mL despite enhanced adherence counseling. To evaluate adherence, caregivers were asked to return drug at all scheduled visits. Percentage of medication return was calculated and poor adherence was defined as 20% greater than expected medication return for each drug.

### Statistical analysis

All outcomes were compared between sexes within treatment strata as well as between treatment groups within sex strata. Intent-to-treat comparisons were made for treatment groups. Kaplan-Meier methods and log-rank tests were used to describe time to virologic failure. For comparisons between groups at specific time points, we used the Wilcoxon rank-sum test for non-normally distributed continuous variables, t-test for normally-distributed continuous variables, and Chi-square or Fisher exact tests for categorical variables. Generalized estimating equation models were used to compare outcomes with repeated measurements over time using a first order autoregressive correlation structure, adjusting for baseline differences. Tests for interaction between sex and treatment group were performed. All *p*-values are 2-tailed and *p*-values <0.05 were considered statistically significant. Analyses were performed using SAS version 9.1.3 (Cary, North Carolina, USA).

## Results

### Study population

**Figure 1:**
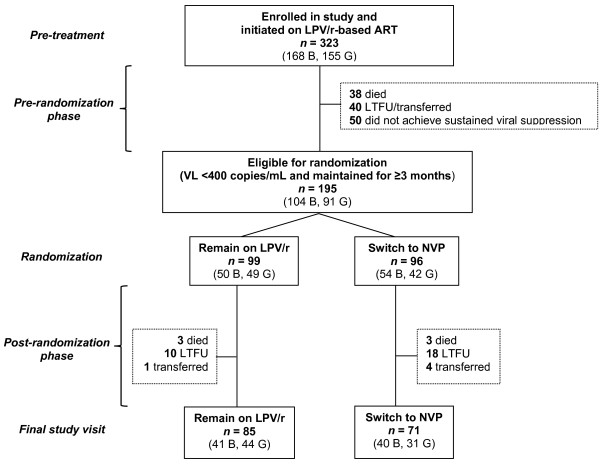
**Flow diagram of study participants in Neverest 2.** Flow diagram of study participants in Neverest 2; Abbreviations: LPV/r, lopinavir-boosted ritonavir; NVP, nevirapine; ART, antiretroviral therapy; LTFU, lost to follow up; B, boys; G, girls.

The flow of participants is shown in Figure 1. A total of 323 HIV-infected children, including 168 (52%) boys, were enrolled in the study and initiated LPV/r-based ART. In the period between initiating LPV/r-based treatment and randomization to either remain on LPV/r or switch to nevirapine (pre-randomization phase), 38 (11.8%) died, 40 (12.4%) did not remain in follow up, and 50 (15.5%) did not meet criteria for randomization. Of the 195 children randomized in the study, 99 remained on LPV/r, including 50 (50.5%) boys, and 96 were switched to nevirapine, including 54 (56.3%) boys. Children were followed for 76 weeks post-randomization during which time four (2.1%) children died and 15 (7.7%) children were lost to follow up. Long-term follow up continued for a minimum of 99 weeks and maximum of 245 weeks after randomization. At study completion in June 2010, six (3.1%) children died, 28 (14.4%) were lost to follow-up, and five (2.6%) transferred out. 156 (80%) completed the final study visit.

### Pretreatment characteristics

The pretreatment characteristics of the 323 children initiating LPV/r-based ART are presented in Table 1. Overall, more boys initiated ART after 6 months of age (*p =* 0.009). Boys also had a lower mean WAZ and HAZ and a greater proportion of boys were underweight and stunted. The pretreatment anthropometric differences remained after adjusting for age at treatment initiation. No differences between sexes were seen in other pretreatment characteristics. In the pre-randomization period, similar proportions of boys and girls died, were lost to follow up, or did not meet criteria for randomization (data not shown). Results were similar if only the 195 children randomized were analyzed, but the mean WAZ of boys and the proportion of boys underweight and stunted were no longer significantly different than girls.

**Table 1:** Pretreatment characteristics of 323 perinatally HIV-infected South African children enrolled in Neverest 2 by sex

**Characteristics**	**Enrolled children (n = 323)**		
	**Boys (n = 168)**	**Girls (n = 155)**	***P*-value**
Age at treatment start, N (%)			
<6 months	41 (24.4)	61 (39.4)	
6-12 months	70 (41.7)	45 (29.0)	**0.009**
12-24 months	57 (33.9)	49 (31.6)	
Median (range) in months	9.6 (2.2-24.2)	7.9 (2.0-24.9)	0.098
HIV-1 RNA quantity, N (%)			0.475
<100,000 copies/ml	16 (10.6)	9 (6.8)	
100,000-750,000 copies/ml	39 (25.8)	39 (29.6)	
≥750,000 copies/ml	96 (63.6)	84 (63.6)	
CD4 count in cells/mm^3^, Mean (SD)	1001 (724)	1011 (786)	0.909
CD4 percent, N (%)			
<10	30 (19.1)	25 (17.0)	
10-14.9	35 (22.3)	26 (17.7)	0.463
≥15	92 (58.6)	96 (65.3)	
Median (range)	16.8 (0.8-43.0)	19.1 (1.2-39.1)	0.163
WHO Stage, N (%)			
I/II	29 (21.0)	22 (17.5)	0.465
III/IV	109 (79.0)	104 (82.5)	
Weight for age Z-score			
Mean (SD)	−2.70 (1.8)	−2.28 (1.6)	**0.041**
Z-score < −2, N (%)	98 (66.7)	71 (49.7)	**0.003**
Height for age Z-score			
Mean (SD)	−3.72 (1.8)	−3.11 (1.6)	**0.003**
Z-score < −2, N (%)	125 (86.2)	108 (76.6)	**0.037**

**Note:** Categorical variables were compared across groups using X^2^ tests; median age, CD4 percentage, and time on therapy were compared using Wilcoxon tests; CD4 count and height and weight for age z-scores were compared using t-tests. Denominators are as shown.

**Abbreviations:***LPV/r* Lopinavir-boosted ritonavir, *NVP* Nevirapine, *ART* Antiretroviral therapy, *LTFU* Lost to follow up.

### Viral load

There were no differences between boys and girls in either the proportion who attained viral load suppression to <400 copies/mL by 6 months into the pre-randomization phase (78.9 vs. 76.9%, *p =* 0.747), or the proportion with viral load <50 copies/mL at randomization (61.5 vs. 64.8%, *p =* 0.634) when all children were on LPV/r. The probability of virologic failure (confirmed viral load >1000 copies/mL) by 52 weeks post-randomization was similar between boys and girls who remained on LPV/r (0 vs. 0.043, *p =* 0.162) as well as between boys and girls who switched to nevirapine (0.224 vs. 0.172, *p =* 0.575). The probability of virologic failure by 156 weeks was also similar between boys and girls who remained on LPV/r (0.115 vs. 0.104, *p =* 0.996) as well as between boys and girls who switched to nevirapine (0.240 vs. 0.238, *p =* 0.954). Proportions of boys and girls in viremia categories (<50, 50–1000, >1000 copies/mL) within treatment groups were similar at all scheduled post-randomization visits.

### CD4 percentage and count

**Figure 2:**
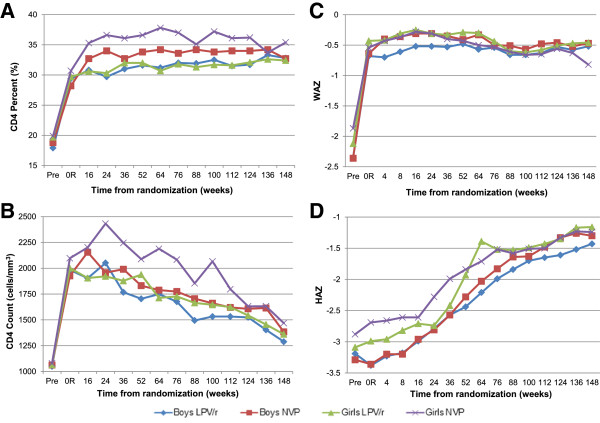
**Immunologic and anthropometric outcomes over time.** Mean CD4 percent **(A)** and CD4 count **(B)**, weight-for-age z-score (WAZ) **(C)** and height-for-age z-score (HAZ) **(D)** before treatment, at randomization, and at scheduled visits with measurements through 148 weeks after randomization in Neverest 2 by sex and randomization group; Abbreviations: WAZ, weight-for-age z-score; HAZ, height-for-age z-score; LPV/r, lopinavir-boosted ritonavir; NVP, nevirapine.

While the mean CD4 percentage and cell count increased in the pre-randomization phase for boys and girls in both treatment groups, differences in CD4 response between sexes were noted among those switched to nevirapine post-randomization. Girls who switched to nevirapine had a more robust CD4 count response than boys switched to nevirapine from 24 weeks up to 112 weeks post randomization (on average CD4 count 306 cells/μL higher, *p =* 0.045), with significantly higher mean CD4 cell counts at 24, 64, and 100 weeks post-randomization (*p =* 0.027, 0.036, 0.043 respectively) and higher CD4 percentage at 64, 76, and 100 weeks post-randomization (*p =* 0.028, 0.035, 0.047, respectively) (Figure 2). After adjustment for age at ART initiation, pretreatment WAZ, HAZ, and CD4 count, on average CD4 count remained significantly higher for girls than boys from 24 to 112 weeks after randomization to nevirapine (p = 0.0089). The observed differences were no longer detectable at 148 weeks.

### Metabolic and laboratory assessments

No significant differences in mean total cholesterol, LDL, HDL, and triglycerides were found pretreatment, at randomization, or at scheduled visits 36, 88, and 136 weeks post-randomization between boys and girls (data not shown) [23]. Upon exit from the study, girls continuing on LPV/r had a higher total cholesterol:HDL ratio and lower mean HDL concentration than boys on LPV/r, as well as compared to girls on nevirapine (3.8 vs. 3.0, *p =* 0.002 and 48 vs. 57 mg/dL, *p =* 0.007, respectively), and girls on LPV/r had a higher mean HOMA-IR than boys on LPV/r (Table 2). There were no differences in proportions of boys and girls within treatment groups with drug related rashes or Grade 3 or 4 ALT at any scheduled post-randomization visits (data not shown).

**Table 2:** Metabolic and other laboratory assessments of 156 perinatally HIV-infected South African children at the final study visit of Neverest 2 by sex within treatment randomization group

**Measurement**		**LPV/r (n = 85)**			**NVP (n = 71)**		
		**Boys (n = 41)**	**Girls (n = 44)**	***P*-value**	**Boys (n = 40)**	**Girls (n = 31)**	***P*-value**
**Total Cholesterol,** mg/dL	Mean (SD)	170 (39)	172 (39)	0.816	163 (25)	159 (38)	0.533
Acceptable: <170	N (%)	24 (58.5)	21 (47.7)	0.603	29 (72.5)	23 (74.2)	0.884
Borderline: 170-199		10 (24.4)	14 (31.8)		8 (20.0)	5 (16.1)	
Elevated: ≥200		7 (17.1)	9 (20.5)		3 (7.5)	3 (9.7)	
**HDL,** mg/dL	Mean (SD)	54 (14)	48 (12)	**0.044**	60 (15)	57 (16)	0.352
Abnormal: <35	N (%)	2 (4.9)	5 (11.4)	0.277	0 (0.0)	2 (6.4)	0.103
Normal: ≥35		39 (95.1)	39 (88.6)		40 (100.0)	29 (93.6)	
**LDL,** mg/dL	Mean (SD)	98 (31)	101 (38)	0.664	89 (24)	87 (29)	0.738
Acceptable: <110	N (%)	28 (68.3)	28 (63.6)	0.895	31 (77.5)	25 (80.7)	0.516
Borderline: 110-129		7 (17.1)	9 (20.5)		7 (17.5)	3 (9.7)	
Elevated: ≥130		6 (14.6)	7 (15.9)		2 (0.5)	3 (9.7)	
**Total Cholesterol: HDL Ratio**	Mean (SD)	3.3 (1.0)	3.8 (1.1)	**0.049**	2.8 (0.7)	3.0 (1.0)	0.547
**Triglycerides,** mg/dL	Mean (SD)	88 (34)	99 (42)	0.207	69 (27)	74 (32)	0.457
Normal: ≤150	N (%)	37 (90.2)	37 (84.1)	0.398	39 (97.5)	30 (96.8)	0.855
Abnormal: >150		4 (9.8)	7 (15.9)		1 (2.5)	1 (3.2)	
**C-Reactive Protein,** mg/L	Mean (SD)	3.2 (5.6)	3.8 (6.5)	0.453	9.5 (23.8)	9.8 (18.1)	0.423
Normal: ≤3	N (%)	33 (80.5)	36 (81.8)	0.875	27 (67.5)	19 (61.3)	0.587
Elevated: >3		8 (19.5)	8 (18.2)		13 (32.5)	12 (38.7)	
**Glucose,** mg/dL	Mean (SD)	96.9 (7.1)	96.4 (7.8)	0.736	97.6 (9.2)	93.7 (8.5)	0.070
Normal: <110	N (%)	80 (98.8)	41 (100.0)	0.332	74 (98.7)	39 (97.5)	0.375
Impaired: ≥110		1 (1.2)	0 (0.0)		1 (1.3)	1 (2.5)	
**HOMA-IR**	Mean (SD)	0.90 (0.5)	1.16 (0.6)	**0.036**	1.17 (1.0)	0.96 (0.6)	0.289
Normal: ≤3.16	N (%)	41 (100.0)	44 (100.0)	1.0	36 (92.3)	31 (100.0)	0.115
Resistance: >3.16		0 (0.0)	0 (0.0)		3 (7.7)	0 (0.0)	

**Abbreviations:***LPV/r* Lopinavir-boosted ritonavir, *NVP* Nevirapine, *HDL* High-density lipoprotein, *LDL* Low-density lipoprotein, *HOMA-IR* Homeostatic model assessment of insulin resistance.

### Anthropometrics

WAZ for boys and girls increased rapidly in the pre-randomization phase (Figure 2); the mean change in WAZ for boys was greater than girls, though not significant (1.69 vs. 1.50, *p =* 0.386). After randomization, boys on LPV/r consistently had a lower WAZ than boys on nevirapine through 148 weeks, but this difference was not statistically significant. There were no sex differences in likelihood of dropping more than one Z score in WAZ before 52 weeks post-randomization.

Mean change in HAZ was greater for girls than boys in the pre-randomization phase when all children were on LPV/r, though this difference was not significant (0.14 vs. -0.06, *p =* 0.473). After randomization, height for boys and girls on both treatment regimens improved through 148 weeks (Figure 2). Mean HAZ was significantly lower for boys than girls on nevirapine at randomization (−3.38 vs. -2.99, *p =* 0.021). HAZ stayed lower for boys on nevirapine than girls on nevirapine through 52 weeks post-randomization (b = −0.57, *p =* 0.031). The association remained when adjusted for age at initiation of ART (b = −0.54, *p =* 0.026), but not when adjusted for pretreatment HAZ (b = −0.38, *p =* 0.145).

At the final study visit, girls had a larger %BF by BIA than boys (18.7 vs. 12.8%, *p <* 0.0001). A similar pattern was observed by cumulative skinfold sum, though this difference was not significant (42.9 vs. 39.7 mm, *p =* 0.076). Patterns of fat were similar between boys and girls in both treatment randomization groups (Table 3). There were no differences in proportions of children with lipodystrophy between boys and girls.

**Table 3:** Body composition measures of 156 perinatally HIV-infected South African children at the final study visit of Neverest 2 by sex within treatment randomization group, Mean (SD)

**Measurement**	**LPV/r (n = 85)**			**NVP (n = 71)**		
	**Boys (n = 41)**	**Girls (n = 44)**	***P*-value**	**Boys (n = 40)**	**Girls (n = 31)**	***P*-value**
MUAC, cm	16.1 (15.2)	15.5 (2.0)	0.288	15.2 (1.8)	15.1 (2.1)	0.844
MWC: MHC Ratio	1.25 (1.9)	0.93 (0.1)	0.287	0.95 (0.1)	0.95 (0.1)	0.773
SFS, mm	41.9 (12.1)	44.0 (10.1)	0.408	37.2 (9.7)	41.3 (10.3)	0.120
%BF by BIA	14.5 (6.2)	19.2 (7.0)	**0.002**	10.9 (7.6)	17.9 (6.9)	**0.0002**
**Extremity Fat Area**						
Arm Fat Area, cm^2^	5.52 (2.0)	6.05 (2.2)	0.259	4.91 (1.3)	5.65 (2.0)	0.063
% of Fat in Arm	26.6 (5.5)	31.5 (9.6)	**0.006**	27.2 (8.2)	30.9 (8.2)	0.063
Leg Fat Area, cm^2^	14.9 (5.5)	16.5 (6.5)	0.236	12.3 (4.4)	15.2 (6.1)	**0.025**
% of Fat in Leg	20.1 (5.6)	23.4 (7.4)	**0.028**	17.7 (4.5)	21.6 (6.4)	**0.006**
**Regional Fat Proportion of Total Body Fat**						
Arm: [(BSF + TSF)/SFS]	0.30 (0.05)	0.30 (0.04)	0.985	0.31 (0.05)	0.29 (0.03)	0.197
Trunk: [(SSF + SISF + USF)/SFS]	0.46 (0.07)	0.45 (0.06)	0.484	0.47 (0.05)	0.47 (0.05)	0.658
Leg: [MTSF/SFS]	0.24 (0.04)	0.25 (0.04)	0.297	0.22 (0.03)	0.24 (0.04)	**0.026**
**Trunk-Extremity Skinfold Ratios**						
Trunk-Arm: [(SSF + SISF)/(BSF + TSF + SSF + SISF)]	0.49 (0.06)	0.50 (0.06)	0.652	0.50 (0.04)	0.51 (0.06)	0.306
Trunk-Leg: [(SSF + SISF)/(MTSF + SSF + SISF)]	0.55 (0.06)	0.55 (0.07)	0.978	0.58 (0.04)	0.57 (0.06)	0.278

**Abbreviations:***LPV/r* Lopinavir-boosted ritonavir, *NVP* Nevirapine, *MUAC* Mid-upper arm circumference, *MWC* Mid-waist circumference, *MHC* Maximum hip circumference, *BSF* Bicep skinfold, *TSF* Tricep skinfold, *SSF* Subscapular skinfold, *SISF* Suprailiac skinfold, *USF* Umbilical skinfold, *MTSF* Mid-thigh skinfold, *SFS* Skinfold sum, %*BF* Percent body fat, *BIA* Bioimpedance analysis.

**Note:** Cumulative skinfold sum (SFS) was calculated by adding BSF, TSF, SSF, SISF, USF, and MTSF. Extremity fat areas, regional fat proportions of total body fat, and trunk-extremity skinfold ratios were calculated as previously reported [22].

### Drug concentrations and adherence

Of those randomized to remain on LPV/r, the plasma concentrations of ritonavir adjusted for WAZ and time since dose was on average 0.115 μg/ml higher in girls than boys (*p =* 0.048) in scheduled post-randomization visits. No significant sex differences in lopinavir concentrations were observed. Of those switched to nevirapine, there were no sex differences in nevirapine concentrations. There were no differences between boys and girls in the proportion of children non-adherent to their drugs at any post-randomization scheduled visits as assessed by medication return percentages.

## Discussion

In this randomized clinical trial, we assessed sex differences in treatment outcomes of South African HIV-infected children initiated on LPV/r before age two and later randomized to remain on LPV/r or switch to nevirapine after attaining suppression of viremia. Though no sex differences were observed in virologic response, girls switched to nevirapine had a more robust immunologic response than boys switched to nevirapine. In addition, after a minimum of 99 and maximum of 245 weeks on ART, girls on LPV/r had a higher total cholesterol:HDL ratio and lower mean HDL concentration than boys on LPV/r. As children remain on life-long treatment, these sex disparities noted early in life may have implications for long term clinical outcomes.

Few pediatric studies have assessed sex differences in virologic response to ART. Similar to a recent meta-analysis of virologic outcomes in treatment-experienced adults [3] and a randomized trial comparing nevirapine and LPV/r-based treatment initiation in young children with no prior nevirapine exposure [29], our study observed no sex differences in suppression of virus in response to ART. This finding is in contrast to an earlier study in children on mono- or dual-nucleoside analogue therapy that reported lower viral load levels in girls than boys [16].

Our finding that girls switched to nevirapine have a greater immunologic response than boys is similar to results from two observational studies conducted in children receiving non-nucleoside reverse transcriptase inhibitors (NNRTI)-based regimens [17,18]. A stronger CD4 cell count response in women, compared to men after treatment initiation, has also been reported in a large cohort study of HIV-infected adults, the majority of whom were initiated on NNRTI-based therapy [30], but these results appear to be explained by higher baseline CD4 count in women. In contrast, our association remained when adjusted for pretreatment CD4 count as well as age at ART initiation. The reason for the difference in CD4 response by sex among children randomly assigned to switch to nevirapine is unclear, but it does not appear to be due to better drug adherence or viral suppression or differences in drug concentrations; the difference may reflect underlying sex differences in CD4 parameters that have been reported in healthy uninfected children [31]. The consequences of this time-limited difference are also unclear. High baseline CD4 cell count has been identified as a risk factor for nevirapine-related hepatotoxicity during ART initiation in women and pregnant women [32], but we did not see increased abnormal ALT levels or drug-related rashes in girls on nevirapine. Although it is unclear if the incremental increase in CD4 response we observed among girls switched to nevirapine will have long term benefits, the simultaneous rapid improvement in growth suggests that there is a clinical benefit for these girls during this time period.

Although studies in adults observe more lipid abnormalities in ART-treated women [11,33-36], to our knowledge, no prior studies have reported sex differences in metabolic outcomes in children. The differences we observe do not appear to be explained by underlying sex differences in healthy children [37,38] and may be a result of greater exposure to ritonavir in girls. Ritonavir is known to adversely affect lipid metabolism through suppression of the degradation of the nuclear form of sterol regulatory element binding proteins in the liver and other sites, resulting in increased fatty acid and cholesterol production [39]. Higher ritonavir drug concentration have been previously reported in adult women [40], and may be driven by sex differences in body size and composition as well as drug metabolism, transport, and excretion [13].

Sex differences in growth parameters during ART were also detected. Though height remained significantly lower for boys on nevirapine than girls on nevirapine up to 52 weeks post-randomization after adjusting for age at initiation of ART, it was no longer significant when adjusted for pretreatment height. We have previously reported that earlier age of ART initiation can affect growth [41]. Few studies have compared growth responses between sexes; one study of older children reported higher rates of stunting in boys than girls [42]. In our study, we noted higher levels of fat in girls than boys which may reflect underlying sex differences in total body fat observed in healthy children [43-45]. Greater fat accumulation in girls, as reported in studies of older HIV-infected children during puberty, may be related to estrogen effects on fat accumulation [46,47]. In the present study in which all children were pre-pubertal, however, we did not observe sex differences in prevalence of lipohypertrophy or other forms of lipodystrophy [46,48].

Although our study is one of the first to evaluate sex differences in childhood response to potent combination ART, it has limitations. Children in this trial met eligibility criteria for treatment based on South African guidelines in place at the time and only those who attained viral suppression in the first year of therapy were randomized [49]. Thus, our study has limited generalizability to populations with suboptimal adherence or virologic response. In addition, we were not able to assess the potential relationship between drug pharmacokinetics and body composition, or detect rare adverse events.

## Conclusions

In this study, we observed no sex differences in viral suppression; however, compared to boys switched to nevirapine, girls switched to nevirapine had a more robust immune recovery. In addition, girls on LPV/r had a more unfavorable lipid profile compared to boys on LPV/r at the final study visit. This study provides a glimpse into early childhood sex differences related to ART. The course and long term consequences of these differences with respect to immunologic and metabolic outcomes is unknown. Future studies of sex differences among HIV-infected children should focus on pharmacokinetics as well as pathophysiologic pathways including chronic inflammation, immune senescence and mitochondrial dysfunction which may influence the therapeutic response and risk of complications. The potential effects of factors such as malnutrition, infections, co-morbidity, viral sub-type, and underlying genetic profiles also warrant further investigation during childhood and adolescence.

## Abbreviations

ALT: Alanine aminotransferase; ART: Antiretroviral treatment; BIA: Bioimpedance analysis; HAZ: Height-for-age z-score; HDL: High-density lipoprotein; HOMA-IR: Homeostasis model assessment of insulin resistance; LPV/r: Ritonavir-boosted lopinavir; LDL: Low-density lipoprotein; NNRTI: Non-nucleoside reverse transcriptase inhibitor; WAZ: Weight-for-age z-score; %BF: Total body fat percent.

## Competing interests

The authors declare that they have no competing interests.

## Authors’ contributions

LK, EJA, and AC designed the study. AC, LM, and RS were responsible for data collection. HM, LW, and SM contributed to the pharmacokinetic analysis. SS, SMA, and LK contributed to analysis of data and interpretation of results. All authors read and approved the final manuscript.

## Pre-publication history

The pre-publication history for this paper can be accessed here:

http://www.biomedcentral.com/1471-2431/14/39/prepub
